# Cryptogenic hepatitis simulating cyst rupture and hydatid jaundice in a patient with preexisting asymptomatic hydatid cyst

**Published:** 2017

**Authors:** Ulas Aday, Cuneyt Kayaalp, Murat Kapan

**Affiliations:** 1 Diyarbakir Egitim Arastirma Hospital, Department of Surgery, Diyarbakir, Turkey.; 2 Inonu University, Liver Transplantation Institute, Malatya, Turkey.; 3 Dicle University, Department of Surgery, Diyarbakir, Turkey.

**Keywords:** Hydatid liver disease, Surgery, Jaundice, Cryptogenic hepatitis

## Abstract

**Background:**

Rupture into the biliary ducts is the most frequent complication of hydatid liver disease. In endemic areas of *Echinococcus granulosus,* development of jaundice in a patient with liver cyst is initially suspected to have hydatid cyst.

**Case Presentation:**

A 48 year-old woman with history of asymptomatic hydatid liver cysts was admitted to the emergency department with right upper quadrant abdominal pain, increased levels of liver enzymes, bilirubin and alkaline phosphatase and the initial clinical diagnosis was the hydatid cyst rupture into the bile ducts. Surgery was planned but radiological evaluation (MRI) revealed non-dilated intra-extra biliary ducts. High suspicion of hydatid rupture required diagnostic ERCP that was normal and surgery was cancelled then. A possible diagnosis of coexistent hepatitis was suspected. Liver function tests normalized gradually and no cyst rupture was determined during surgery.

**Conclusion:**

These findings suggest considering the possible development of cryptogenic hepatitis in patients with preexisting hydatid cyst.

Hydatid disease is a parasitic infection caused by *Echinococcus granulosus* and it is still a serious health problem in endemic areas. Hydatid cysts can originate from all visceral organs but most often is located in the liver. Rupture of cysts into the biliary tract is the most frequent complication (1). Passage of cyst contents into biliary tract results in clinical jaundice and abnormalities in liver function tests (1). This study described a case with similar clinical condition presented with different manifestations.

## Case Presentation

A 48 year-old woman was admitted to our emergency department with right upper quadrant abdominal pain, nausea and vomiting which persisted for one week. She had a history of appendectomy and a known asymptomatic liver hydatid cyst which did not receive any treatment yet. Clinical examination revealed jaundice, and pain at epigastric and right upper quadrant areas, with normal stool color at rectal examination. Laboratory test results demonstrated hypochromic microcytic anemia at hemoglobin level of 11.6 g/dl, the total bilirubin was 4.4 mg/dL, direct bilirubin 3.2 mg/dL, alanine aminotransferase (ALT) 1386 U/L, aspartate aminotransferase (AST) 1071 U/L, alkaline phosphatase (ALP) 307 U/l and gamma glutamyl transpeptidase (GGT) 156 U/L. Abdominal ultrasound showed a 68 mm in diameter exophytic and multivesicular hydatid cyst located at segment II and III of the liver.

**Figure 1:**
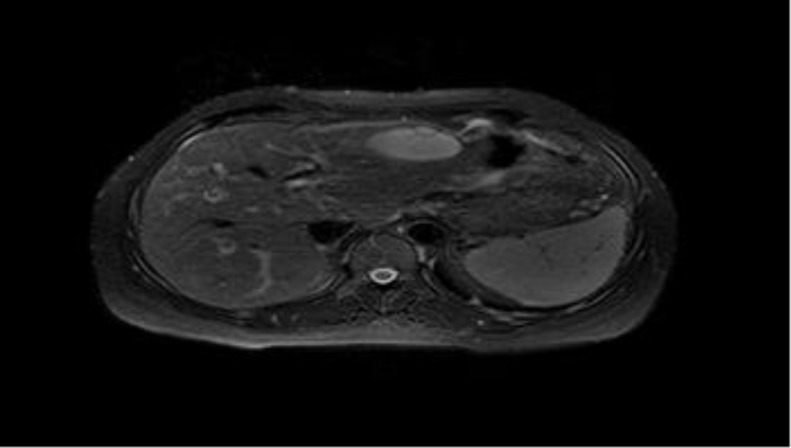
Preoperative magnetic resonance imaging

Gallbladder, biliary tract and pancreas were normal. Abdominal magnetic resonance imaging confirmed sonographic findings without dilation of intrahepatic and extrahepatic biliary ducts (figure 1). 

**Figure 2:**
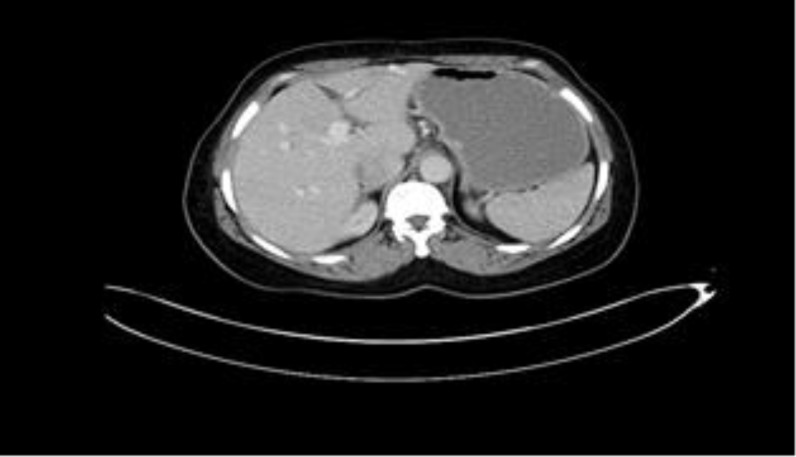
Postoperative computed tomography after six years

Viral hepatitis and tumor markers were negative. She denied using any drugs or toxic products. The initial diagnosis of the patient was hydatid liver cyst ruptured into the biliary tract, hence an endoscopic retrograde cholangiopancreatography (ERCP) was performed with normal findings. The possibility of hepatitis with unknown origin (cryptogenic hepatitis) caused to postpone surgery until three weeks later when the levels of serum bilirubin, ALP and GGT decreased to normal values, but ALT and AST values ​​remained high at 464 U/L and 372 U/L, respectively. The patient underwent an exploratory surgery which found no cyto-biliary rupture. A liver biopsy was performed and the patient was discharged on postoperative day 5. At this time, the ALT and AST values were 175 U/L and 58 U/L, respectively. Histopathology of the hydatid cyst was confirmed. The patient was well in the last follow-up performed 6 years after discharge with normal liver function tests and normal findings in computed tomography scanning (figure 2).

## Discussion

Rupture into bile ducts is the most common and serious complication of hydatid liver disease. The predisposing factors for cysto-biliary communications are; preoperative increased levels of liver enzymes and large size cysts or multiple cysts (2). The symptoms and signs of rupture are dependent to the extent of biliary tract obstruction. Patients with incomplete biliary tract obstructions can be asymptomatic and is diagnosed by altered liver function tests. Development of severe obstruction results in nausea, vomiting, right upper quadrant pain, jaundice and pale stool (1). Direct bilirubin level increases more than indirect bilirubin and ALP and GGT levels increase more suggestively than AST and ALT values. Radiological findings can be obscure if there is an occult cysto-biliary communication. If frank rupture occurs, dilatation of intra and extrahepatic bile ducts is inevitable. The criteria used for diagnosis of hydatid jaundice were *(i)* an existing hydatid liver cyst, *(ii) *elevated bilirubin levels (direct bilirubin increase was dominant) and *(iii) *negative laboratory results for viral hepatitis and tumor markers. Retrospectively, we examined the diagnostic errors and found a clue against the presence of hydatid jaundice. The following findings may be helpful in ruling out hydatid jaundice; *(i)* normal color stool, *(ii) *greater increase in the serum levels of AST-ALT as compared to ALP-GGT and *(iii) *unremarkable dilatation in the bile ducts. 

Hepatocellular jaundice is the injury of hepatocytes without biliary obstruction. The most frequent causes of acute hepatocellular damage are viruses, natural and chemical toxic agents and drugs. Although performing laparotomy is necessary in patients with hydatid cyst rupture, anesthesia and trauma of surgery may cause hepatocellular jaundice to deteriorate, perhaps fatally (3). In the present case, surgery was postponed to avoid these risks.

In the countries where hydatid disease is rare, the differential diagnosis of hydatid jaundice can be difficult as well (4). It may simulate cholecystitis or viral hepatitis (5). Development of cryptogenic hepatitis in patients of endemic areas may result in the misdiagnosis of hydatid jaundice 

## References

[1] Kayaalp C, Blumgart LH, Belghiti RJ, DeMatteo RP (2007). Hydatid cyst of the liver. Surgery of liver, biliary tract and pancreas.

[2] Aday U, Kapan M, Onder A (2011). Liver hydatid cyst associated with biliary tract: Is it an important complication indicator?. J Curr Surg.

[3] Dodgion C, King M, Bewes PC, Cairns J, Thornton J Primary surgery: volume one: non trauma. Online edition, 1999. Chapter 6. Obstructive Jaundice.

[4] Bücker A, Adam G, Truong S, Wein B (1996). Icterus--sometimes something is wrong: the rupture of an echinococcal cyst into the biliary tract. Rofo.

[5] Stuiver PC, Overbosch D, Jongsma CK, Gooszen HG, Smelt AH (1989). Jaundice caused by hydatid disease of the liver. Neth J Med.

